# Environmental Health Research Implications of Methylmercury

**DOI:** 10.1289/ehp.1103580

**Published:** 2011-07-01

**Authors:** Toshihide Tsuda, Takashi Yorifuji, Masazumi Harada

**Affiliations:** Department of Environmental Epidemiology, Okayama University Graduate School of Environmental Science, Okayama, Japan, E-mail: tsudatos@md.okayama-u.ac.jp; Department of Epidemiology, Okayama University Graduate School of Medicine, Dentistry and Pharmaceutical Sciences, Okayama, Japan; Department of Social Welfare Studies, Kumamoto Gakuen University, Kumamoto, Japan

In “Adverse Effects of Methylmercury: Environmental Health Research Implications,” Grandjean et al. (2010) reviewed the scientific discoveries of health risks resulting from methylmercury exposure, including the history of the Minamata disease incident. Although their title states “research implications,” the authors failed to convey some important caveats from the incident.

First, Grandjean et al. (2010) explained the incident as if serious delays of the recognition of “the exact cause (methylmercury)” deferred the corrective action. However, recognition of an etiologic agent is not a necessary condition for prevention (Goodman et al. 1990). When source and transmission are identified, they must be eliminated even if the etiologic agent is unknown. In the case of Minamata disease, even in 1956 when the first patient was identified, eating contaminated seafood was determined to be a cause of the disease; this occurred 3 years before the etiologic agent was identified (Tsuda et al. 2009). Grandjean et al. (2010) cited Harada (2004), who wrote,

However, with no specific causative substance [etiologic agent] determined, there was no legal basis for a ban on fishing. (Under Item 2 of the Food Sanitation Act, it was not possible to prohibit fishing while the cause was undetermined.)

However, in Japan, even with no specific etiologic agent determined, the Food Sanitation Act has routinely been enacted when causal food and/or causal facility was determined.

Second, Grandjean et al. (2010) mentioned the “diagnostic difficulties” of methylmercury poisoning cases. Lack of investigation of the Minamata disease incident as food poisoning resulted in unnecessary diagnostic difficulties; such difficulties do not usually arise in food-poisoning incidents in Japan. In the case of Minamata disease, in 1977 the Japanese Ministry of Environment (JME) established the criteria for diagnosis, which required combinations of signs that were advocated by the JME to be medically correct. However, the truth is that the JME recognized a lack of medical evidence on the criteria [Committee on Research and Human Rights/Japanese Society and Psychiatry and Neurology (CRHR-JSPN) 2003]. Moreover, medical researchers in Japan have pointed out that the criteria were medically incorrect (CRHR-JSPN 1998). The “diagnostic difficulties” may have obscured who was affected and had neurological signs.

Third, Grandjean et al. (2010) stated that, “Only in 2009 was a law enacted to provide compensation to most of the remaining group of victims.” However, it was not compensation. For Minamata disease, unless the affected persons are diagnosed by the above-mentioned criteria, they are not counted as patients and are thus not properly compensated. About 2,200 patients have been diagnosed with Minamata disease and have been compensated, whereas at least several tens of thousands of victims who have neurological signs characteristic of methylmercury poisoning have not been recognized as patients and have not been not properly compensated (McCurry 2006).

Fourth, Grandjean et al. (2010) described the “scientific account” of the cat experiment in 1959, which was published after a 40-year delay (Eto et al. 2001). However, the report provided only pathological findings, and the detailed explanation of the cat experiment had already been published in 1965 (Tomita 1965). The latter would be enough for prevention and control.

Finally, because the JME and local governments have been defendants in Minamata disease lawsuits, research funds from JME and the local governments may affect researchers’ attitudes, possibly causing conflicts of interest.
